# Targeting Mitochondrial Metabolism to Save the Failing Heart

**DOI:** 10.3390/life13041027

**Published:** 2023-04-16

**Authors:** Christina Schenkl, Estelle Heyne, Torsten Doenst, Paul Christian Schulze, Tien Dung Nguyen

**Affiliations:** 1 Department of Cardiothoracic Surgery, Jena University Hospital, Friedrich Schiller University Jena, Am Klinikum 1, 07747 Jena, Germany; 2 Department of Medicine I (Cardiology, Angiology, Critical Care Medicine), Jena University Hospital, Friedrich Schiller University Jena, Am Klinikum 1, 07747 Jena, Germany

**Keywords:** mitochondria, metabolism, heart failure, heart failure treatment

## Abstract

Despite considerable progress in treating cardiac disorders, the prevalence of heart failure (HF) keeps growing, making it a global medical and economic burden. HF is characterized by profound metabolic remodeling, which mostly occurs in the mitochondria. Although it is well established that the failing heart is energy-deficient, the role of mitochondria in the pathophysiology of HF extends beyond the energetic aspects. Changes in substrate oxidation, tricarboxylic acid cycle and the respiratory chain have emerged as key players in regulating myocardial energy homeostasis, Ca^2+^ handling, oxidative stress and inflammation. This work aims to highlight metabolic alterations in the mitochondria and their far-reaching effects on the pathophysiology of HF. Based on this knowledge, we will also discuss potential metabolic approaches to improve cardiac function.

## 1. Introduction

Heart failure (HF) is a condition in which heart function is insufficient to meet the body’s oxygen demand. It is not a specific cardiac disorder but rather a clinical syndrome characterized by increased intracardiac pressure and/ or reduced cardiac output resulting from diverse cardiac abnormalities. Therefore, HF may be the common end stage of numerous cardiovascular diseases, such as coronary artery disease, hypertension, cardiomyopathies, heart valve disease or a combination of these [1].

More than 64 million patients worldwide suffered from HF in 2017 [2]. In developed countries, HF is the most frequent diagnosis in hospitalized patients older than 65 years [3]. The prevalence of HF is expected to increase by 25% within the next 20 years, which is related to population aging and the improved treatment of acute cardiovascular events [4]. Importantly, the 10-year survival rate of HF is only 35% [5], and the prognosis of HF is comparable to that of most cancers [6]. Thus, HF remains a tremendous medical and economic challenge.

A variety of frameworks have been used to define subsets of HF. Based on the clinical course, one can distinguish between acute and chronic HF. Acute HF often presents with a rapid onset and worsening symptoms. In contrast, chronic HF is a progressive condition that develops over a prolonged period of time and usually involves structural remodeling of the myocardium. Depending on the left ventricular ejection fraction (LVEF), HF can be categorized into HF with reduced (≤40%) LVEF (HFrEF), HF with mildly reduced (41–49%) LVEF (HFmrEF) and HF with preserved (≥50%) ejection fraction (HFpEF) [1].

Because of its unspecific definition and overlapping classifications, the term “heart failure” has been used differently among clinicians and bench scientists. Preclinical research has focused on chronic left heart failure with reduced EF. Therefore, data reviewed in this work mostly apply to this classic form of HF. For the sake of simplicity, we will also use the term “heart failure” in its traditional meaning, i.e., conditions with reduced EF.

The last three decades have witnessed significant discoveries in device and pharmacologic therapy for HF. Present medical treatments for HF are centered on inhibiting neurohormonal activity and diminishing fluid retention. However, the mortality and morbidity of HF remain unacceptably high. Furthermore, there are very limited treatment options for HFpEF [1], which has a high and growing prevalence [7]. Thus, approaches beyond the present therapeutic concepts are necessary to move forward in the fight against HF.

Cardiac work requires a large amount of adenosine triphosphate (ATP) for both contraction and relaxation. Since the ATP reserve of the heart is limited, cardiac function hinges on the competence of mitochondrial ATP supply. HF has been linked to impaired myocardial energetics characterized by decreases in ATP content, phosphocreatine/ATP ratio and ATP flux through creatine kinase [8,9]. It is, therefore, not surprising to see perturbations at the level of mitochondria. This work reviews the key mechanisms contributing to mitochondrial dysfunction and energy deficiency in HF. We previously demonstrated that metabolic remodeling in HF also involves biosynthetic and regulatory pathways [10]. Accordingly, we will illustrate, herein, how mitochondrial changes are linked to central dysregulations in HF, such as oxidative stress, inflammation and Ca^2+^ mishandling. Based on this knowledge, we will discuss strategies targeting mitochondrial function, which may hold promise in the development of novel drugs for HF.

## 2. HF Is Characterized by Mitochondrial Dysfunction

### 2.1. Decreased Substrate Oxidation

Apart from the postprandial phase, the oxidation of fatty acids (FAs) provides roughly 70% to 90% of cardiac ATP, while the remaining ATP is generated from the oxidation of glucose, lactate, ketone bodies and amino acids. Myocardial FA oxidation undergoes marked changes during the development of HF. In compensated hypertrophy, it has been found to be preserved [11,12,13] or decreased [14,15,16]. However, in advanced HF, that is, when systolic dysfunction is manifest, there is consistent evidence that genes of β-oxidation enzymes (e.g., medium and long-chain acyl-coenzyme A dehydrogenase—MCAD and LCAD) are downregulated [17,18], and FA oxidation is markedly reduced [14,19,20,21].

Impairments in cardiac FA oxidation have been linked to changes in regulators. Reduced gene expression and activity of the mitochondrial FA transporters, carnitine palmitoyltransferase (CPT) 1 or 2, have been shown in rodents, dogs and humans with HF [13,14,22,23,24]. CPTs are induced by the peroxisome proliferator-activated receptors (PPARs). In cardiomyocytes undergoing hypertrophic growth, Barger et al. demonstrated reduced activity of PPARα accompanied by low expression of CPT 1 [25]. The protein levels of PPARα in left ventricular biopsies obtained from patients with end-stage HF were found to be decreased by approximately 50% when compared to control donor hearts [26]. FA utilization may also be stimulated by estrogen-related receptor α (ERRα) and peroxisome proliferator-activated receptor gamma coactivator 1-α (PGC-1α), which are key regulators of mitochondrial biogenesis [27]. We and others found lower gene expression of these proteins along with reduced FA oxidation rates in rats and mice with HF induced by pressure overload [15,28,29]. Likewise, DNA microarray analysis of failing human hearts also revealed a synchronized reduction in the expression of PGC-1α and ERRα target genes [30].

The healthy heart barely uses glucose for ATP generation under normal conditions but can raise glucose oxidation to maintain energy supply when FA oxidation is reduced, for instance, during postprandial state or hypoxia [31,32]. In the development of HF, however, glucose utilization is also affected. We demonstrated, in rats subjected to pressure overload, that cardiac glucose oxidation tended to increase initially but remained unchanged during compensated hypertrophy, and it finally decreased when systolic dysfunction was present [14]. However, we found unchanged glucose oxidation in infarcted rat hearts despite systolic dysfunction [23]. Therefore, changes in cardiac glucose utilization in HF are inconsistent [10] and may depend on the stage and cause of HF. Regardless of the transient changes in glucose oxidation, because FA oxidation produces more ATP than glucose oxidation, it is unlikely that impaired FA oxidation in the failing myocardium is adequately compensated for by glucose utilization.

The mechanisms contributing to impaired glucose oxidation in HF are manifold. Although rates of glucose uptake in the hypertrophied and failing heart are elevated, glucose is increasingly channeled into biosynthetic and regulatory pathways, such as the pentose phosphate pathway, the hexosamine biosynthetic pathway, as well as the glycerolipid synthesis pathway. The reader is referred to our previous work for details on these cytosolic changes [33]. At the mitochondrial level, we and others found decreased activity of the pyruvate dehydrogenase (PDH) complex [12,13,34], possibly resulting from its inhibition by branched-chain amino acids (BCAAs) [35]. Importantly, pyruvate carboxylation is activated, leading to further suppression of glucose oxidation via substrate competition [36,37].

### 2.2. Perturbation of the Tricarboxylic Acid (TCA) Cycle

The intactness of the TCA cycle is essential for oxidative phosphorylation and contractile function [38]. Because intermediates of the TCA cycle are also consumed by anabolic reactions (cataplerosis) [39,40], they need to be constantly replenished by anaplerotic pathways to maintain cycle activity. In the failing myocardium, the abundance of most TCA cycle intermediates is reduced [41], suggesting an imbalance between these two antagonistic processes. Before systolic dysfunction is manifest, the heart undergoes ventricular remodeling in terms of hypertrophy and fibrosis. Hypertrophic growth requires metabolic adaptations of cardiomyocytes that recruit amino acids for de novo protein biosynthesis. Specifically, proteolytic mechanisms, such as autophagy and proteasome, are activated [42,43] while the degradation of amino acids, a central anaplerotic pathway, is inhibited [41,44]. Although anaplerosis via pyruvate carboxylation is enhanced [36,37], data from stable isotope tracing suggest that glucose derived from this pathway is also directed to the synthesis of nucleotides and proteins [45,46]. Therefore, the depletion of the TCA pool most likely results from a shift toward cataplerosis to promote cardiac hypertrophy.

### 2.3. Impaired Oxidative Phosphorylation

In HF, diminished oxidative phosphorylation rates have consistently been shown in animal models of infarction [47,48], ischemia [49], rapid pacing [50] or pressure overload [28,29,51,52,53,54,55]. These findings are in line with observations in patients with systolic dysfunction [20,56]. Impaired mitochondrial oxidative phosphorylation may result from decreased activities of complexes I and III, as demonstrated both in animal models [57,58,59] and in patients with reduced ejection fraction [60,61]. While a sufficient supply of fatty acids, glucose and amino acids are crucial for ATP generation, micronutrients, including coenzyme Q10, zinc, copper, selenium and iron, may also have a strong impact on the functionality of the respiratory complexes. This issue has been extensively reviewed elsewhere [62]. Defects or dysfunction of respiratory complexes may promote hypertrophic remodeling and HF, as shown in mice with genetic inhibition of complex I [63].

### 2.4. Impaired Mitochondrial Biogenesis

Competent mitochondrial biogenesis is essential to preserve the abundance and integrity of mitochondria. Data from animal models of HF [28] and humans [60,64,65] have identified reduced ventricular mitochondrial content, suggesting a perturbation in mitochondrial biogenesis. This process is regulated by a set of nuclear-encoded transcription factors, including ERRα, nuclear respiratory factors (NRFs) and mitochondrial transcription factor A (TFAM). Their transcriptional activities are coordinately regulated by PGC-1α and its homologue PGC-1β, which have overlapping targets and may support each other [27]. In keeping with the notion of impaired mitochondrial biogenesis, we and others found reduced expressions of PGC-1α/β, ERRα and TFAM in failing rodent hearts induced by pressure overload [28,29,55]. Importantly, a lack of PGC-1α or PGC-1β may accelerate the development of HF in mice subjected to pressure overload [29,66]. Therefore, disruption of mitochondrial biogenesis in HF may be attributed to impaired PGC-1α/β signaling and potentially facilitate the progression of contractile dysfunction.

### 2.5. Altered Morphology and Stability

In addition to the reduction in mitochondrial content, mitochondria in the failing myocardium are characterized by structural abnormalities. In failing rat hearts induced by pressure overload, we found marked disorganization of mitochondrial cristae [28]. In patients with HFpEF and HFrEF due to pressure overload or ischemic heart disease, Chaanine et al. found mitochondrial fragmentation, cristae destruction and reduced mitochondrial area [60]. In addition, mitochondria in HF may have a deranged inner structure with a loss of mitochondrial granules and electron-dense matrix and degeneration of the vacuole [60]. Mitochondrial membranes are characterized by a unique phospholipid pool containing the dimeric phospholipid cardiolipin, which regulates cristae morphology and structure as well as mitochondrial supercomplex assembly [67,68]. Cardiolipin may play a role in preserving mitochondrial structure and function in HF because animal models of HF and explanted failing human hearts consistently showed reduced cardiolipin amount [69,70,71].

### 2.6. Disturbances in Mitochondrial Dynamics

Mitochondrial fusion and fission are dynamic processes governing mitochondrial network structure. They interact with each other to adapt global mitochondrial activity to metabolic demand and, therefore, have a great impact on cellular energetics [72]. There is accumulating evidence suggesting an involvement of mitochondrial dynamics in the pathophysiology of HF. In failing rat and human hearts, Chen et al. found small and fragmented mitochondria associated with decreased protein expression of the mitochondrial fusion protein Optic Atrophy 1 (OPA1) [73]. Similarly, Sabbah et al. reported a consistent downregulation of fusion-related proteins, including Mitofusin 2 (Mfn2) and OPA1 [74], in ventricular samples of dogs and humans with HF. Importantly, mice lacking OPA1 showed increased reactive oxygen species (ROS), reduced mitochondrial DNA (mtDNA) copy number [75] and developed more severe ventricular hypertrophy and systolic dysfunction following transverse aortic constriction [76].

Fission, the dispersal of mitochondrial networks, is the initial step to sort out damaged mitochondria to be eliminated by mitophagy [77,78]. Mitochondrial fission occurs upon translocation of cytosolic dynamin-related protein (Drp-1) to the mitochondria. There are mounting data suggesting elevated mitochondrial fission in animal models of pressure overload, myocardial infarction and doxorubicin-induced cardiomyopathy [79,80,81]. In sum, mitochondrial dynamics in HF are unbalanced, characterized both by a reduction in fusion and an increase in fission activity. Because these processes are responsive to metabolic cues, it is tempting to assume underlying metabolic mechanisms, which are yet to be identified.

## 3. Consequences of Mitochondrial Dysfunction

### 3.1. Energy Depletion

Although data on cardiac energetics in pathological hypertrophy have been inconclusive, there is consistent evidence of decreased myocardial ATP availability in advanced HF [10]. The mechanisms leading to ATP depletion are manifold but undoubtedly involve multiple derangements in mitochondrial metabolism and regulation, as illustrated in the previous section. Myocardial energy shortage not only compromises relaxation and contraction directly but also affects other critical ATP-dependent processes such as ion transport, including Ca^2+^ handling (see below) and biosynthesis of cellular components. With the progression of ventricular remodeling (e.g., stiffening, dilatation, mitral regurgitation), this energy deficiency may become critical for contractile function because the efficiency of transduction from ATP to mechanical power decreases. It is, therefore, not surprising that cardiac energy status may predict outcome in patients with HF [82].

### 3.2. Impaired Calcium Handling

Intact Ca^2+^ homeostasis is essential for both systolic and diastolic function. Myocardial contraction requires a transient increase in cytosolic Ca^2+^ concentration, facilitating the formation of cross-bridges between actin and myosin. For cardiac relaxation, Ca^2+^ is sequestered from the cytoplasm via reuptake into the sarcoplasmic reticulum through the sarcoplasmic–endoplasmic reticulum Ca^2+^ ATPase (SERCA) and, to a lesser extent, by extrusion to the extracellular space by the Na^+^/Ca^2+^ exchanger (NCX) and the sarcolemmal Ca^2+^ ATPase [83]. In addition to excitation–contraction coupling, Ca^2+^ is involved in other important cell functions by acting as a ubiquitous second messenger [84,85,86].

In HF, there is ample evidence for defective Ca^2+^ handling, whose mechanisms are incompletely understood [87,88,89]. Mitochondria are key regulators of Ca^2+^ homeostasis. They not only provide ATP to drive Ca^2+^ pumps but also possess several Ca^2+^ transport channels, allowing for mitochondrial Ca^2+^ influx and efflux [90,91], which prevents drastic intracellular Ca^2+^ oscillations. Energy depletion due to mitochondrial dysfunction in HF may worsen the activity of SERCA and, therefore, disrupt Ca^2+^ cycling. On the other hand, impaired Ca^2+^ homeostasis may also affect mitochondrial function [86]. Although mitochondria have a high buffer capacity for Ca^2+^, excessive accumulation of mitochondrial Ca^2+^, as seen in HF [92], causes mitochondrial damage. Mitochondrial Ca^2+^ overload, which is attributable to increased activity of mitochondrial Ca^2+^ uniporter (MCU) [93,94], Ca^2+^/calmodulin-dependent protein kinase II (CaMKII) [95,96] or to decreased NCX expression [88], may impair oxidative phosphorylation (OXPHOS), promote mitochondrial ROS generation and trigger mitochondrial permeability transition pore (mPTP) opening, eventually leading to cell death [97,98,99]. In summary, mitochondrial dysfunction potentially results in defective Ca^2+^ homeostasis, which, in turn, further deteriorates mitochondrial function to form a vitious cycle that impairs both systolic and diastolic function.

### 3.3. Oxidative Stress

Cellular redox state is determined by production of reactive oxygen species (ROS) and activities of antioxidant enzymes. Important sources of ROS include mitochondria, xanthine oxidase and NADPH oxidase (NOX) [100,101,102]. By contrast, antioxidative defense mostly relies on NADPH-dependent glutathione reductase, thioredoxin, manganese superoxide dismutase (MnSOD) and catalase [103]. Depending on the concentration as well as their origin, ROS may play a physiological or pathological role. While a certain level of ROS is essential to maintain cardiac function [104,105], high levels of ROS have been associated with ventricular dysfunction in animal models of HF [106,107] and in patients [64,108,109,110] with HF.

Mitochondrial dysfunction has been identified as a major contributor to oxidative stress in HF. Defects in complex I, specifically in mitochondrially encoded subunits, may promote mitochondrial ROS production [111]. In HF induced by rapid pacing [112] or pressure overload [57], ROS originating from complex I were elevated. Further potential mitochondrial sources of ROS in heart failure include complex III [57,113] or complex II [114,115], potentially through reverse electron flow to complex I [116]. It has been proposed that inhibition of complex V due to increased protein acetylation may also be involved in ROS production in HF [117]. These data indicate a strong interrelationship between mitochondrial respiratory complex dysfunction and ROS production in HF. Once mitochondrial ROS production and, thus, cytosolic ROS are increased, this may potentiate ROS production from neighboring mitochondria through a process termed “ROS induced ROS release” [118]. One of the first consequences of increased ROS seems to be damage to mtDNA, which further disturbs respiratory chain function (complex I dysfunction) and results in a vicious cycle [111,112].

ROS levels may also be affected by disturbances in mitochondrial Ca^2+^ handling. In a murine model of myocardial infarction-induced HF, mitochondrial Ca^2+^ overload resulting from diastolic sarcoplasmatic reticulum Ca^2+^ leaks increased ROS production, which, in turn, further increased Ca^2+^ leaks, mitochondrial dysfunction and compromised cardiac function [92]. ROS may affect Ca^2+^ homeostasis by modifying SERCA2 [119] or the Ca^2+^ channel RyR [120]. However, because both increased and decreased mitochondrial Ca^2+^ levels have been associated with mitochondrial ROS [53,121,122,123,124], the relationship between mitochondrial Ca^2+^ handling and ROS remains uncertain.

Numerous alterations in metabolic pathways have also been associated with oxidative stress in HF. Sun et al. demonstrated, in isolated mitochondria, that elevated BCAAs induce complex I-dependent mitochondrial respiratory inhibition and, consequently, increase ROS production in a dose-dependent manner [125]. In addition to the augmented generation of ROS, myocardial antioxidative defense may be impaired in HF as a consequence of reduced MnSOD activity [126] or decreased NADH/NAD+ redox potential [123,127] or depletion of alpha-ketoglutarate [128]. Decreased glutathione reduction due to the lack of NADPH represents another important cause of oxidative stress. NADPH production may be affected by protein carbonylation of nicotinamide nucleotide transhydrogenase [129]. Furthermore, NADPH may be overconsumed by pyruvate carboxylation via malic enzyme, as demonstrated in pressure-overloaded rat hearts [37,130]. Consistently, suppression of malic enzyme in these hypertrophied hearts restored GSH (reduced glutathione) content and enhanced cardiac performance [130].

Oxidative stress has been related to key features of structural remodeling in HF. ROS are potent mediators of cardiac hypertrophy by activating numerous hypertrophic signaling cascades, including extracellular signal-regulated kinases (ERK)1/2, c-Jun N-terminal kinase (JNK), p38 mitogen-activated protein kinase (MAPK), protein kinase B (Akt) [131], calcineurin, nuclear factor kappa-light-chain-enhancer of activated B cells (NF-κB) and tyrosine kinases [132]. Furthermore, ROS may be involved in myocardial fibrosis and dilatation by affecting the extracellular matrix. In rats with pressure-overload-induced HF, NOX2 activation increased expression of procollagen I and III and interstitial fibrosis [133]. Excessive ROS levels can decrease the flexibility of titin [134], activating matrix metalloproteinases (MMPs) and lowering the levels of inhibitors of MMPs, known as tissue inhibitor of metalloprotease-1 (TIMP) [135]. As a result, these processes may trigger pathological hypertrophy, myocardial stiffness and ventricular dilation, leading to diastolic and systolic dysfunction.

### 3.4. Inflammation

The development of HF is accompanied by inflammatory processes. A hallmark of HF is the elevation of pro-inflammatory cytokines, such as tumor necrosis factor α (TNF-α), interleukin (IL)-1β and IL-6 [136]. Notably, increased TNF-α altered myocardial Ca^2+^-handling in isolated myocytes [137], and TNF-α overexpression in mice was sufficient to induce HF [138], suggesting a direct involvement of inflammation in the pathophysiology of HF. In line with this notion, TNF-α neutralizing antibody was able to enhance cardiac function in a transgenic mouse model of HF [139]. Furthermore, targeted anti-cytokine therapy with a monoclonal antibody against IL-1β improved HF outcomes in patients [140].

Several metabolic perturbations in the hypertrophied or failing heart have been linked to inflammation. Defective myocardial FA oxidation results in an accumulation of medium- and long-chain acylcarnitines. Interestingly, serum levels of acylcarnitines are elevated in HF [141] and they have been associated with adverse clinical outcomes [142]. Although evidence for cardiac effects of acylcarnitines in the settings of HF is still lacking, they have been shown to activate proinflammatory signaling pathways [143] and promote oxidative stress and insulin resistance in various in vitro models [144]. In addition, the accumulation of BCAA owing to defective myocardial amino acid catabolism may also provoke oxidative stress and inflammation [125,145,146]. Further potential contributors to myocardial inflammation in HF include oxidative stress and mtDNA leakage. Disturbed oxidative phosphorylation can raise ROS levels, which, in turn, can facilitate the assembly of NLRP3 inflammasomes and the release of proinflammatory cytokines [147]. Additionally, mtDNA may stimulate the innate immune system causing inflammation in various tissues [148], which may be explained by its potential bacterial origin. To prevent the release of mtDNA into the cytosol, damaged mitochondria are eliminated by autophagy. However, although mitophagy is activated in pathological hypertrophy, this process is likely disturbed because mtDNA leakage is increased, which may induce Toll-like receptor (TLR) 9-mediated inflammatory responses and dilated cardiomyopathy [149].

The detailed mechanisms of how metabolic and mitochondrial changes in cardiomyocytes induce myocardial inflammation remain elusive because this process involves highly complex interactions among various cardiac and immune cells. Regardless of the initiating mechanisms, the terminal common pathway of the inflammatory cascade encompasses the migration of neutrophils and macrophages into the myocardium [150,151]. By clearing dead cells and renewing the extracellular matrix, these cells primarily contribute to myocardial tissue repair. However, persistent activation of neutrophils may evoke further myocardial damage by releasing ROS, granular components and pro-inflammatory mediators [152]. Furthermore, M2-like macrophages may induce myocardial fibrosis by activating cardiac fibroblast and myofibroblast or by autonomous production of collagen [153,154], which finally worsens both diastolic and systolic function.

**Figure 1:**
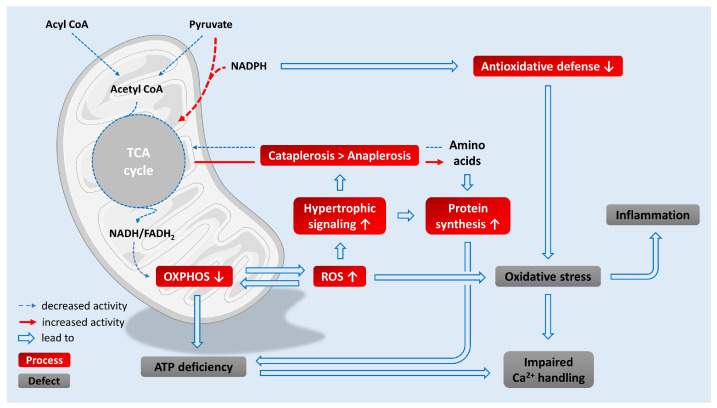
**Proposed mechanisms linking mitochondrial metabolic changes to major defects in the failing heart.** The oxidation of both fatty acids and pyruvate is impaired. There is also a shift towards cataplerosis to recruit amino acids for hypertrophic growth. Both changes result in depletion of TCA cycle pool. Furthermore, OXPHOS is disturbed, and ATP is increasingly consumed by protein synthesis. All these maladaptations culminate in ATP deficiency. Perturbations in OXPHOS foster mitochondrial ROS production. The carboxylation of pyruvate consumes NADPH and therefore impedes antioxidative defense. The resulting oxidative stress promotes inflammation and impairs Ca^2+^ handling. Ca^2+^ homeostasis may also be affected by ATP deficiency.

Figure 1 depicts key metabolic mechanisms linking mitochondria to major pathological processes in the failing myocardium. For the sake of clarity, some potential mechanisms are omitted in the illustration and only described in the text.

## 4. Potential Therapeutic Approaches

By causing myocardial energy depletion, oxidative stress, inflammation and impaired Ca^2+^ handling, mitochondrial dysfunction plays a central role in the pathophysiology of HF. The following section discusses therapeutic strategies targeting mitochondrial metabolism to counteract these key perturbations and improve cardiac function.

### 4.1. Stimulation of Fatty Acid Oxidation

Over the last thirty years, there has been extensive research on using metabolic treatments to treat heart diseases, with a particular focus on cardiac FA utilization. However, there is no agreement on how myocardial FA oxidation should be modified in HF. While some researchers have suggested inhibiting FA oxidation as a potential metabolic strategy for treating HF [155,156], there is an increasing body of evidence that challenges this viewpoint. Studies on rodent models of pressure overload, myocardial infarction or genetic cardiomyopathy have shown protective effects associated with high-fat diets [157]. Additionally, stimulating myocardial FA oxidation has been found to preserve cardiac function in pressure overload [158,159] or myocardial infarction [160]. Because the transport of most fatty acids into the mitochondria matrix is dependent on L-carnitine, stimulating L-carnitine is crucial for the maintenance of FA oxidation. In a hypertensive rat model of HFpEF, L-carnitine supplementation reduced ventricular fibrosis, prevented pulmonary congestion and improved survival [161]. In addition to stimulating uptake and oxidation of fatty acids, L-carnitine may also increase glucose oxidation by stimulating pyruvate dehydrogenase in conditions of high levels of free FAs [162]. In our systematic review and meta-analysis, we found that approaches that promote cardiac FA oxidation significantly improve heart function while those that lower FA oxidation showed no effects [163].

### 4.2. Stimulation of Glucose Oxidation

Pathological hypertrophy has been associated with a redirection of glucose towards biosynthetic and regulatory pathways [33], which may limit the ability of glucose oxidation to compensate for the reduction in FA oxidation. To improve cardiac energetics, it may, therefore, be beneficial to stimulate glucose oxidation through the PDH complex. The most investigated PDH stimulator is dichloroacetate (DCA). DCA treatment reduced ventricular hypertrophy and improved cardiac function and survival in Dahl salt-sensitive rats with hypertension [12]. The observed effects were associated with enhanced flux through the pentose phosphate pathways, increased energy reserves and less oxidative damage and apoptosis [12]. DCA also improved postischemic cardiac output in hypertrophied rats [164]. Our meta-analysis revealed that approaches increasing cardiac glucose oxidation significantly enhance ventricular function, while those inhibiting glucose oxidation are detrimental [163]. While there is compelling preclinical evidence supporting the advantages of improving myocardial glucose oxidation, the clinical significance of this metabolic approach is yet to be determined. Data on short-term effects of DCA in HF patients are inconsistent [165,166] and long-term data are lacking, possibly because of the risk of peripheral neuropathy in chronic treatment [167]. There is a need for novel drugs that target myocardial glucose oxidation. In a previous study, we found that treatment with glucagon-like peptide-1 (GLP-1) improved diastolic function and increased survival rates in a rat model of HFpEF. Notably, these effects were accompanied by a preservation of cardiac glucose oxidation. Our findings not only imply that GLP-1 may boost glucose oxidation in the failing myocardium but also suggest enhancing cardiac glucose oxidation for HFpEF [168].

### 4.3. Stimulation of Ketone Body Oxidation

Ketone bodies may serve as additional or alternative substrates for the heart. In HF, increased myocardial utilization of ketone bodies has been consistently observed [169,170,171]. Since inhibition of ketone body oxidation in pressure-overloaded mouse hearts aggravated ventricular dysfunction [172,173], this change most likely represents a necessary metabolic adaptation to maintain ATP production. In keeping with this notion, ventricular remodeling and dysfunction in mice subjected to transverse aortic constriction and in canines with pacing-induced cardiomyopathy were ameliorated by feeding with a ketogenic diet [173]. It is noteworthy that sodium–glucose cotransporter-2 (SGLT2) inhibitors, which have been established as potent drugs for treating both HFrEF and HFpEF, are also associated with increased ketone body utilization. While ketone bodies may enhance the energy status of the failing heart by acting as alternative substrates, potential mechanisms underlying their cardioprotective effects have been discovered. For instance, in a mouse model of HFpEF, raising β-hydroxybutyrate levels reduced the formation of NLPR3 inflammasomes, mitochondrial hyperacetylation and dysfunction, as well as myocardial fibrosis [174]. Considering the effects of ketone bodies on multiple pathways, including histone deacetylation and redox signaling [175,176], additional mechanisms are likely but remain to be evaluated in the context of HF.

### 4.4. Modulation of Anaplerotic Pathways

As illustrated above, the TCA cycle pool is depleted, potentially resulting from an increased recruitment of amino acids for hypertrophic growth. Restoring the activity of the TCA cycle can be achieved by supplementing anaplerotic substrates. Inhibition of BCAA catabolism, which generates acetyl CoA and propionyl CoA, occurs in the failing heart [125]. Although activation of BCAA catabolism has been associated with reduced oxidative stress [125] and inflammation [146], the enrichment of the TCA cycle with acetyl CoA and propionyl CoA may also contribute to its therapeutic effects. Both acetyl CoA and propionyl CoA can be supplied by odd-chain ketone bodies. We previously showed that feeding rats triheptanoin, which provides C5-ketone bodies to the heart, resulted in increased cardiac glucose oxidation and reduced ventricular hypertrophy and diastolic dysfunction following aortic constriction [177]. Similarly, supplementation of alpha-ketoglutarate in mice with aortic constriction ameliorated left ventricular hypertrophy, fibrosis and systolic dysfunction [178].

Another method to maintain cardiac anaplerotic activity is to provide the heart with exogenous amino acids. At first glance, this concept seems to contradict the benefits of activating BCAA catabolism. Nevertheless, if the downregulation of amino acid catabolism in the failing heart reflects its higher demand for amino acids, providing exogenous amino acids may prevent this maladaptation and retain anaplerosis. In line with this hypothesis, cardioprotection following amino acid supplementation has been reported in rats [179] and patients [180,181,182] with HF.

### 4.5. Targeting Mitochondrial Biogenesis, Morphology and Dynamics

Because PGC-1α signaling, which governs mitochondrial biogenesis and FA utilization, is downregulated in HF, activation of this pathway has been considered a promising approach to protect mitochondrial and heart function. However, studies have failed to demonstrate functional benefits of overexpressing PGC-1α in mice with aortic constriction [183,184]. Interestingly, overexpression of TFAM, a downstream signal of PGC-1α, reduced LV remodeling and preserved cardiac function in mice with myocardial infarction [185]. TFAM may protect mtDNA and mitigate Ca^2+^ mishandling and excessive ROS production. The import of exogenous TFAM with the help of TFAM-packed exosomes might represent one feasible way to improve mitochondrial biogenesis and function in HF [186].

Other interventions target mitochondrial morphology and dynamics. Pharmacologic inhibition of poly(ADP-ribose) polymerase (PARP) prevented mitochondrial fragmentation, increased mitochondria size and cristae density and prevented left ventricular hypertrophy in spontaneous hypertensive rats [187]. Efforts to restore mitochondrial phospholipid content by administration of exogenous cardiolipin were successful in cells [188]. However, similar attempts failed in a knock-down mouse model mimicking Barth syndrome with cardiomyopathy [188]. In mice with pressure overload-induced HF, treatment with the mitochondrial division inhibitor (Mdivi) decreased ventricular fibrosis and preserved cardiac function [79]. Finally, treatment with berberine activated mitophagy via the PINK1/Parkin pathway and preserved ventricular function in mice subjected to pressure overload [189].

## 5. Conclusions

Mitochondrial dysfunction is a key feature of HF. It is characterized by multiple disturbances at the levels of substrate oxidation, TCA cycle, oxidative phosphorylation as well as defects in mitochondrial biogenesis, structure and dynamics. Mitochondrial dysfunction results in energy shortage directly compromising contractile work and other ATP-consuming processes, such as chemical synthesis and ion transport. Furthermore, several metabolic changes in mitochondria may additionally depress heart function by provoking oxidative stress, inflammation and Ca^2+^ mishandling. By acknowledging the central role of mitochondria in the pathophysiology of HF, we propose that improving mitochondrial function would yield substantial benefit by mitigating several harmful processes synchronously and should, therefore, be the focus of therapeutic research.

## Data Availability

Not applicable.
